# Partial Characterization of Novel Bacteriocin SF1 Produced by *Shigella flexneri* and Their Lethal Activity on Members of Gut Microbiota

**DOI:** 10.1155/2019/6747190

**Published:** 2019-05-06

**Authors:** Carlos Padilla, Verónica Carrasco-Sánchez, Andrés Padilla, Olga Lobos

**Affiliations:** ^1^Departamento de Microbiología, Facultad de Ciencias de la Salud, Universidad de Talca, Talca 3460000, Chile; ^2^Servicio de Medicina Interna, Hospital Regional de Talca, Talca 3460000, Chile

## Abstract

A strain of *Shigella flexneri* producing bacteriocin was isolated from a patient with diarrhea. The main objective of this study was to isolate and partially characterize the bacteriocin. The producing microorganism was identified using biochemical, serological, and molecular methods. The lethal activity of the *S. flexneri* strain was studied using the drop method. This bacterial strain showed activity against different strains of *E. coli* and *B. fragilis*. Using immunological techniques, it was determined that *S. flexneri* belongs to serotype 2a, and by PCR, the presence of the ipaH plasmid was determined. By chromatographic techniques, it was determined that the bacteriocin is a peptide of high purity with a molecular weight of 66294.094 Da. The amino acid composition and sequence were determined by the Edman reaction, and a sequence of 619 amino acid residues was obtained. Only in five positions of this sequence, the amino acid glutamine changed to glutamic acid with respect to colicin U produced by *S. boydii*. From an ecological point of view, it could be assumed that SF1 bacteriocin contributes to eliminate some members of the normal microbiota of the human intestine, facilitating colonization and then producing the invasion process that characterizes the pathogenicity of *Shigella*.

## 1. Introduction

Bacteriocins are proteins or antimicrobial peptides produced by different bacterial species, which have a broad or narrow spectrum of lethal action [1, 2]. Usually, these products exert their antagonistic role on other bacterial species by competing for the same ecological niche [3].

In vitro investigations related to the detection and characterization of bacteriocins showed that the biosynthesis is altered by various physical factors. Furthermore, it has been proposed that the production of bacteriocins can be induced by unfavorable conditions of bacterial growth or chemical agents such as mitomycin C [4–6].

Some members of the Enterobacteriaceae family have genetic determinants that encode bacteriocins, whose frequently are located in plasmids [2]. These antibacterial products show a broad spectrum of action and have a variable molecular weight range between 25 and 80 kDa (colicins) or below 9 kDa (microcins) [7, 8]. *E. coli* is noted for its colicin production, very similar to the products synthesized by *S. sonnei*, *S. boydii*, and *S. dysenteriae* [8]. A few studies show bacteriocinogenic activity produced by *S. flexneri*. Preliminary studies demonstrated for the first time the bacteriogenic activity of *S. flexneri* on *E. coli* and *B. fragilis* strains isolated from feces of healthy humans [9, 10].

*Shigella* is the most common etiologic agent of dysenteric diarrhea. The species of this genus have the ability to invade and multiply in the human intestinal epithelium, causing an acute inflammatory response and tissue destruction. The infection usually spreads from person to person through the faecal-oral route, and a very small inoculum (10–100 bacterial cells) is enough to cause disease [8].

Previous studies demonstrated the presence of one strain of *S. flexneri* with antibacterial capacity. In addition, it was reported that the antibacterial product is a bacteriocin with antagonistic activity on *E. coli* and *B. fragilis* [9]. In order to deepen the knowledge about this interesting antibacterial substance, the main objective of this study was to purify and perform a partial characterization of the bacteriocin produced by the *S. flexneri* strain.

## 2. Materials and Methods

### 2.1. Bacterial Strains and Antimicrobial Spectrum of Bacteriocin SF1

A bacteriocin-producer strain of *S. flexneri* was isolated from a patient of 31 years of age with dysenteric diarrhea in the Regional Hospital of Talca, Chile. The patient signed an informed consent for the use of the isolated strain. The bacterium was identified using microbiological and biochemical methods described in Bergey's Manual [11].

Furthermore, serological identification was performed by means of an agglutination test with polyvalent and monovalent serum for somatic O antigen (Denka Seiken, Japan) according to the manufacturer's instructions. The Scientific Ethics Committee of University of Talca approved this study.

As target strains of the lethal action of the bacteriocin SF1, different bacterial species belonging to the Department of Microbiology of Universidad de Talca were used. The target strains tested were *E. coli*, *S. boydii*, *S. sonnei*, *Salmonella typhi*, *S. typhimurium*, *Enterobacter agglomerans*, *Streptococcus pyogenes*, *Klebsiella pneumoniae*, *Citrobacter freundii*, *Morganella morganii*, *Providencia stuartii*, *Bacillus subtilis*, *Staphylococcus aureus*, *Listeria monocytogenes*, and *B. fragilis*. Three strains were tested from each bacterial species. All target strains were grown in Tryptic Soy Broth (Merck, Darmstadt, Germany) at 37°C for 24 h until the early exponential growth phase (OD 0.4 at 600 nm in UV visible spectrophotometer Shimadzu, Japan) except for *B. fragilis*, which was grown on Agar Base Blood (Merck) supplemented with vitamin K1 and hemin in an anaerobic jar (Genbox Anaer Biomerieux, France). Subsequently, this culture was collected and diluted in distilled water until the same OD already mentioned was reached. After that, the target strains were grown on Mueller–Hinton agar (Merck, Germany), except *B. fragilis*, which was sown in the same anaerobic medium already mentioned and incubated in an anaerobic system. On the contrary, the bacteriocinogenic *S. flexneri* strain was cultivated overnight in the Tryptic Soy Broth and subsequently centrifuged to 10,000*g* for 20 min. The antibacterial activity of *S. flexneri* was determined using the drop method. Specifically, all the dishes with the target strains were dried for 10 min at 37°C, and then 5 *μ*L of the *S. flexneri* supernatant was spotted on the lawn [9]. The dishes were incubated at 37°C for 5 h, and then the inhibitory zones were observed.

### 2.2. Molecular Identification of *S. flexneri*

*S. flexneri* was grown in Tryptic Soy Broth (Merck) for 12 h at 37°C. Bacterial genomic DNA was extracted by the MasterPure™ DNA Purification Kit (Epicentre, USA) following the manufacturer's instructions. The concentration of the extracted DNA in solution was determined spectrophotometrically at a wavelength of 260 nm. The purity was measured at 280 nm as previously described [12]. DNA integrity of the template was tested on agarose gel 1% (w/v) and stained with GelRed (Biotium Inc., USA). A PCR assay was performed in a final volume of 25 *μ*L, with a reaction mixture containing 0.25 *μ*g/*μ*L of template DNA, 50 pmol of each oligonucleotide sequence, 1 X PCR master mix, and DNAse-RNAse free distilled water. The primers IpaH-F 5′-CCTTGACCGCCTTTCCGATA-3′ and IpaH-R 5′-CAGCCACCCTCTGAGGTACT-3′ [13, 14] were used. The amplifications were performed using a DNA Engine Thermal Cycler (Laboratories BioRad, USA). PCR conditions were as follows: initial denaturation at 94°C for 2 min, followed by 35 cycles of denaturation at 94°C for 1 min, annealing of oligonucleotide sequences at 62°C for 1 min, and extension at 72°C for 2 min. This was followed by a final incubation for 10 min at 72°C and maintained at 4°C. In addition, a negative control without template DNA was used. PCR-amplified DNA fragments were separated by electrophoresis on 1.5% agarose gel. Furthermore, a wide-range molecular weight marker ladder 100 bp (Invitrogen, Waltham, Massachusetts, USA) was used as standard. The band was stained with GelRed (Biotium Inc). PCR products were visualized and images captured using a Gel Documentation System Doc 1000 (Laboratories BioRad).

### 2.3. Partial Purification of Bacteriocin SF1

*S. flexneri* strain was grown in 500 mL BHI broth at 37°C for 24 h. Subsequently, the culture was centrifuged at 14,000*g* for 35 min at 4°C. The supernatant was treated by a progressive addition of ammonium sulfate to reach 30% saturation. The mixture was kept overnight at 4°C with constant stirring. Then, it was centrifuged at 10,000*g* for 30 min at 4°C. The precipitate was suspended with 50 mM Tris-HCL (pH 8.0) buffer. The resulting supernatant was adjusted to 95% saturation with ammonium sulfate, as described above.

Both samples were centrifuged at 10,000*g* for 30 min at 4°C, and the final pellet containing the bacteriocin SF1 was suspended in a minimal volume of 50 mM Tris-HCL (pH 8.0). The obtained products were dialyzed separately in a MEMBRA-CEL™ MC-18 (Viskase, USA) membrane at 4°C for 48 h in the same buffer used above. The resulting dialysate was loaded onto a column of ion exchange FPLC (fast performance liquid chromatography) using a Mono-Q™ 5/50 GL (GE Healthcare, Sweden) column previously equilibrated with 50 mM Tris-HCL (pH 8.0) and eluted with the same buffer using a gradient of 0 to 1.0 M NaCl in Tris-HCl (pH 8.0). Two mL aliquots were collected and tested to determine antimicrobial activity. Active fractions were mixed, concentrated, and lyophilized. Subsequently, filtration chromatography was performed on FPLC gel using a Superose 12 HR column 10/30 (GE Healthcare Life Sciences, UK) equilibrated with 50 mM Tris-HCl (pH 8.0) and 0.2 M NaCl; the samples were eluted with the same buffer. Active aliquots were processed in HPLC (high-performance liquid chromatography) using a LiChroCART® C18 column (250 × 4.0 mm) (Merck). The mobile phase was 0.1% (v/v) trifluoroacetic acid (TFA) and the solution of 80% (v/v) aqueous acetonitrile containing 0.1% (v/v) of TFA. Aliquots were assayed for detecting the bacteriocin activity. All those positive activity fractions were pooled, lyophilized, and suspended in Milli-Q®, obtaining the partially purified bacteriocin [15].

For all assays, bacteriocinogenic activity related with processes of purification and partial characterization, *E. coli* (EC-7) strain which has a high sensitivity to the bacteriocin studied, was used. This *E. coli* strain was the one that showed the highest sensitivity to bacteriocin among all the studied strains.

### 2.4. Molecular Weight Determination of Bacteriocin SF1

The molecular weight of the bacteriocin SF1 was determined by glycine SDS-PAGE with 5% stacking gel and 12% separating gel using the molecular weight standard Strep Tag II Perfect Protein (Merck) [16]. The gel was stained with Coomassie blue R-250 and washed at room temperature with a solution of acetic acid 5% to remove excess of stain.

### 2.5. Effect of Enzyme Action, pH, and Temperature on Bacteriocin SF1

The bacteriocin SF1 was diluted 5 times in buffer with each enzyme (Sigma, USA) analyzed. The final enzyme concentration was 1 mg/mL. All enzymes were sterilized by filtration through a membrane filter of 0.22 *μ*m (Merck, Germany). For assays, trypsin, *α*-chymotrypsin, and pepsin in a buffer Tris-HCl 20 mM pH 8.0; proteinase K in buffer 20 mM Tris-HCl pH 7.2; and papain in 50 mM phosphate buffer pH 7.0 were used. The enzyme activity was determined by incubating each sample at 37°C for 30 min, 1, and 4 h. Untreated bacteriocin samples were used as controls.

The bacteriocin activity was assessed at different pH values. The pH of the bacteriocin sterilized by filtration was adjusted using the following buffers: KCl-HCl (pH 2.0 and 3.0), acetate (pH 4.0 and 5.0), citrate (pH 6.0), and Tris-HCl (pH 7.0, 8.0, 9.0, 10.0, 11.0, and 12.0). The bacteriocin was diluted twice in different buffers. The resulting mixtures were incubated at 37°C for 30 min. The assay previously described was then performed to detect bacteriocinogenic activity.

On the contrary, the bacteriocin was treated at −76, 4, 25, 37, 60, and 80°C for 30 min as well as 100 and 121°C for 15 min. After the treatment, the samples were diluted 1 : 2, 1 : 4, and 1 : 10 and kept at 4°C for 2 h. Later, tests were performed to determine the biological activity as described above.

### 2.6. Analysis of Antimicrobial Activity in Polyacrylamide Gel under Nondenaturing Conditions

The bacteriocins SF1 was run in a polyacrylamide gel 7.5%. It was subsequently washed with distilled water and placed in a sterile Petri dish. Then, it was covered with a thin mixture of 0.8% Brain Heart Infusion Agar (BHI) (Merck) and 10^4^ CFU/mL *E. coli* EC-7. After, the plate was incubated for 5 h at 37°C for observing the inhibitory zone.

### 2.7. Amino Acid Composition and Sequence Analysis of Bacteriocin SF1

The lyophilized partially purified bacteriocin SF1 was used to obtain the amino acid composition, and the sequence was determined by the Edman reaction in an automated sequencer PPSQ-31A (Shimadzu, Japan) [17].

## 3. Results

### 3.1. Identification of the *S. flexneri* Strain

Bacteriological identification of the *S. flexneri* strain was performed by biochemical and serological methods. The strain belongs to serotype 2a. On the contrary, the molecular identification showed the presence of plasmid 606 bp ipaH, confirming that the strain studied is *S. flexneri*.

### 3.2. Partial Purification of Bacteriocin SF1

The specific activity of bacteriocin SF1 was increased 23 times during the purification process; sixty-nine percent of the antimicrobial activity was recovered (Table 1).

**Figure 1:**
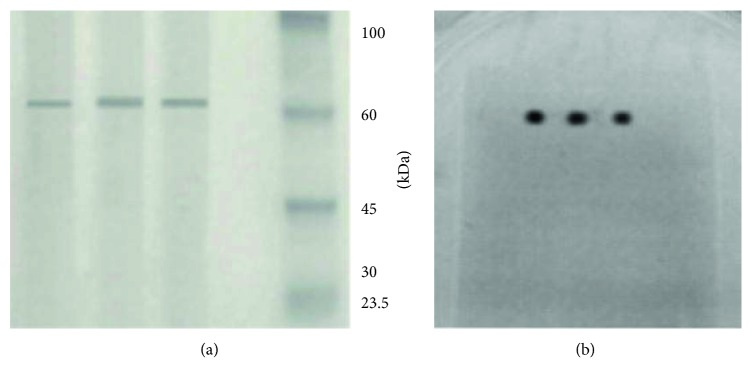
SDS-PAGE and nondenaturing gel: (a) SDS-PAGE, bacteriocin SF1 in triplicate and standard molecular weight Strep Tag II Perfect Protein; (b) antimicrobial activity assay of the bacteriocin SF1 for triplicate.

The SDS-PAGE analysis showed a band of approximately 66 kDa in a triplicate assay (Figure 1(a)). The antibacterial activity analysis showed the presence of an inhibitory zone at the same level of the detected band (Figure 1(b)). The chromatogram of purified bacteriocin SF1 was obtained by HPLC (see Figure S1 in the Supplementary Material for analysis of purity of bacteriocin SF1).

### 3.3. Antimicrobial Spectrum of Bacteriocin SF1

The antimicrobial spectrum of the bacteriocin SF1 was determined on different Gram positive and negative species. The bacteriocin was active only against the three target strains of *E. coli* and *B. fragilis* tested in this research (Table 2).

### 3.4. Effect of Enzymes, pH, and Temperature on Bacteriocin SF1

Only the enzymes proteinase K and papain inactivated the bacteriocin SF1. It was observed that the bacteriocin SF1 lost biological activity only when exposed to 100°C and 121°C. Moreover, the alkaline pH inhibited the antibacterial action (see Table S1 in the Supplementary Material for comprehensive analysis of enzymes activity, temperature, and pH on bacteriocin SF1).

### 3.5. Amino Acid Composition and Sequence Analysis of Bacteriocin SF1

**Figure 2:**
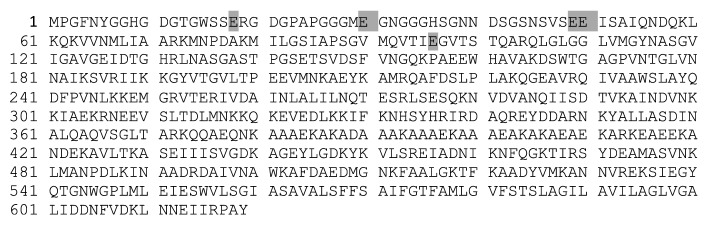
Amino acid sequence of bacteriocin SF1. The observed sequence is similar to the colicin U of *S. boydii*. Only in five positions of the sequence, the amino acid glutamine (Q) changes to glutamic acid (E) (grey highlighted).

The bacteriocin SF1 obtained from *S. flexneri* strain showed a sequence of 619 amino acid residues (Figure 2). Its amino acid composition is shown in Table 3. Its molecular weight was 66294,094 Da.

## 4. Discussion

Species of the genus *Shigella* are among the bacterial pathogens most frequently isolated from patients with diarrhea. Five to fifteen percent of all diarrheal episodes worldwide can be attributed to an infection with *Shigella*, including 1.1 million fatal cases [18, 19].

In this research, the novel bacteriocin produced by the *S. flexneri* strain was named SF1. This substance was sensitive to treatment with proteolytic enzymes, particularly proteinase K and papain, showing their peptidic nature. According to the results, bacteriocin SF1 maintains antibacterial capacity at 80°C, but not at 100°C. The thermolability at 100 and 121°C of this antagonistic substance is also consistent with its chemical composition and corroborates data reported by other authors who have demonstrated that colicins produced by *Shigella* are generally heat labile [7, 20]. By SDS-PAGE, a single band was obtained for bacteriocin SF1. This band was taken and used to detect the protein purity by HPLC. Also, it is important to note that the band in the gel in nondenaturing conditions was responsible for the antagonistic activity in the biological assay.

It was interesting to observe that the amino acid composition of bacteriocin SF1 is similar to that of colicin U produced by *S. boydii* [21]. The specific results of this research showed a small variation that affected only the proportion of glutamine (amino acid imide) and glutamic acid (amino acid) of bacteriocin SF1 in respect to colicin U. In comparison with bacteriocin SF1, the colicin U shows 38 residues of glutamic acid and 28 of glutamine. This result was confirmed by means of the amino acid sequence performed in this research. The amino acidic variation occurs only at five positions in the sequence in which glutamine are replaced by glutamic acid. The molecular weight calculated for the bacteriocin SF1 is 66294.094 Da, compared to the molecular weight of the colicin U which is 66289.1719 Da. Also, according to the results, it is possible to argue that the bacteriocin of *S. flexneri* could be the product of mutations, explaining the differences detected between colicin U and bacteriocin SF1.

In addition, the bacteriocinogenic *S. flexneri* strain might present a selective advantage during the colonization process and before the development of its invasive capacity.

Thus, the bacteriocinogenic activity of *S. flexneri* against *E. coli* and *B. fragilis* would allow understanding how a low infectious dose of *S. flexneri* is capable of displacing these members of the gut microbiota and prevailing in this ecological niche, facilitating colonization and later starting the invasiveness process [22]. Therefore, the possible role of bacteriocin SF1 as a virulence factor should be studied, and further microbiological and molecular studies on the bacteriocin SF1 are necessary to understand in depth its ecological role.

## 5. Conclusions

A novel bacteriocin of 619 amino acid residues and 66294,094 Da of molecular weight, produced by *S. flexneri*, named bacteriocin SF1 has been for the first time detected and partly purified. Bacteriocin SF1 shows lethal activity on *E. coli* and *B. fragilis*, important members of the normal microbiota of the human gut.

## Figures and Tables

**Table 1:** Partial purification of bacteriocin SF1 of *Shigella flexneri*.

Bacteriocin SF1					
Fraction	Volume (mL)	Total activity	Total absorbance at 280 nm	Specific activity	Yield (%)
I	500	50000	6120	9.1	100
II	5	35000	158	210.8	83
III	158	24000	176	208.3	69

**Table 2:** Antimicrobial spectrum of bacteriocin SF1 against different target strains.

Bacterial species studied^*∗*^	Bacteriocin SF1 activity
*E. coli*	+
*B. fragilis*	+
*S. boydii*	−
*S. sonnei*	−
*S. typhi*	−
*S. typhimurium*	−
*E. agglomerans*	−
*K. pneumoniae*	−
*C. freundii*	−
*M. morganii*	−
*P. stuartii*	−
*B. subtilis*	−
*S. aureus*	−
*L. monocytogenes*	−

^*∗*^Three strains were tested from each bacterial species. +: bacterial species sensitive to bacteriocin; −: bacterial species not sensitive to bacteriocin.

**Table 3:** Amino acid composition of bacteriocin SF1.

Aminoacid name	Symbols	Count
Alanine	A	86
Arginine	R	22
Asparginine	N	42
Aspartic acid	D	35
Cysteine	C	0
Glutamic acid	E	43^*∗*^
Glutamine	Q	23^*∗*^
Glycine	G	54
Histidine	H	6
Isoleucine	I	39
Leucine	L	41
Lysine	K	54
Methionine	M	17
Phenylalanine	F	15
Proline	P	14
Serine	S	42
Threonine	T	22
Tryptophan	W	7
Tyrosine	Y	14
Valine	V	43
Sequence length		619

^*∗*^In comparison with SF1, the colicin U of *S. boydii* showed 38 residues of glutamic acid and 28 of glutamine.

## Data Availability

The experimental data used to support the findings of this study are included within the article.

## Supplementary Materials

Supplementary Materials Figure S1 shows the purity of bacteriocin SF1 by means of HPLC. Active aliquots of bacteriocin SF1 were processed in HPLC using a LiChroCART C18 reverse phase. HPLC: mobile phase A: 0.1% trifluoroacetic acid (TFA); mobile phase B: 80% aqueous acetonitrile solution containing 0.1% TFA; linear gradient 0–100% of B solution in 30 min flow rate 1 mL/min; temperature 35°C; active fraction: 34.6 min retention time. Table S1 shows the enzymes activity, temperature, and pH on bacteriocin SF1. Initially, from the untreated bacteriocin, the arbitrary units per mL (AU/mL) were calculated, estimating 25,600 AU/mL. The arbitrary units were calculated based on the reciprocal of the highest dilution with biological activity and multiplied by 100 (dilution factor). *E. coli* EC-7 was used as target strain of lethal action of the bacteriocin SF1.
