# Supplementary material for: Partial Characterization of Novel Bacteriocin SF1 Produced by Shigella flexneri and Their Lethal Activity on Members of Gut Microbiota
Source: Int J Microbiol. 2019 May 6;2019:6747190. doi: 10.1155/2019/6747190 (PMC6526549; doi:10.1155/2019/6747190)
Supplement: Supplementary Materials — Figure S1 shows the purity of bacteriocin SF1 by means of HPLC. Active aliquots of bacteriocin SF1 were processed in HPLC using a LiChroCART C18 reverse phase. HPLC: mobile phase A: 0.1% trifluoroacetic acid (TFA); mobile phase B: 80% aqueous acetonitrile solution containing 0.1% TFA; linear gradient 0–100% of B solution in 30 min flow rate 1 mL/min; temperature 35°C; active fraction: 34.6 min retention time. Table S1 shows the enzymes activity, temperature, and pH on bacteriocin SF1. Initially, from the untreated bacteriocin, the arbitrary units per mL (AU/mL) were calculated, estimating 25,600 AU/mL. The arbitrary units were calculated based on the reciprocal of the highest dilution with biological activity and multiplied by 100 (dilution factor). E. coli EC-7 was used as target strain of lethal action of the bacteriocin SF1. [file 6747190.f1.docx]

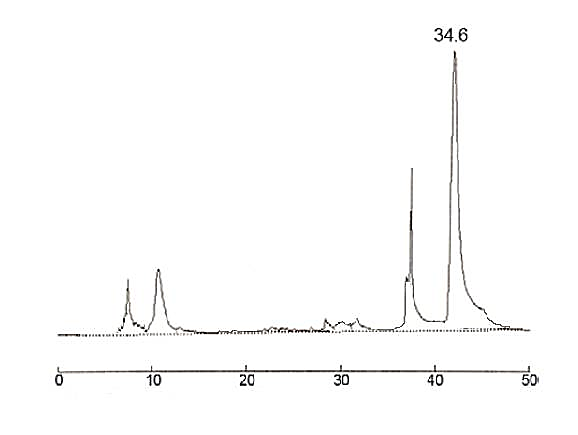


**Figure S1:** LiChroCART C18 reverse phase. HPLC; mobile phase A: 0.1% trifluoroacetic acid (TFA); mobile phase B: 80% aqueous acetonitrile solution containing 0.1% TFA; linear gradient 0-100% of B solution in 30 min flow rate 1 mL/min, temperature 35ºC; active fraction: 34.6 min retention time

Table S1: The effect of enzymes, pH, and temperature on bacteriocin SF1 activity.

SF1 Initial activity

25,600 AU/mL

**Enzime**

Trypsin 12,800

α-chymotrypsin 3,200

Pepsin 3,200

Proteinase K 0

Papain 0

**pH**

2 a 8 12,800

9 a 12 6,400

**Temperature/min**

-76°C/ 30 25,600

4°C/30 25,600

25°C/30 25,600

37°C/30 25,600

60°C/30 25,600

80°C/30 25,600

100°C/10 12,800

121°C/15 12,800
